# A comparative analysis of English for academic purposes teachers’ interactive metadiscourse across the British and Chinese contexts

**DOI:** 10.3389/fpsyg.2022.879713

**Published:** 2022-08-02

**Authors:** Xinxin Wu, He Yang

**Affiliations:** ^1^School of Foreign Languages, Dezhou University, Dezhou, China; ^2^School of Foreign Studies, Jiangnan University, Wuxi, China; ^3^College of Foreign Languages and Cultures, Xiamen University, Xiamen, China

**Keywords:** interactive metadiscourse, comparative analysis, EAP courses, spoken academic genres, discourse community, speech community

## Abstract

This exploratory research compares the interactive metadiscourse use by native English-speaking English for academic purposes (EAP) writing teachers in the United Kingdom and their non-native counterparts in the Chinese contexts. The analysis is based on a self-compiled corpus, including two sub-corpora, which were composed of instructor contributions to classroom discourse: eight sessions of EAP lessons from the Chinese context and eight sessions of EAP lessons from the British context. Adopting an interpersonal model of metadiscourse, the two sub-corpora were compared to examine the similarities and differences in their use of interactive metadiscourse. Findings of the comparative analysis reveal that EAP teachers from both contexts rely heavily on transition markers and frame markers to organize their teaching but differ in particular linguistic realizations. This may indicate the impact of a range of factors such as logical preferences, development order of acquisition, discourse community, and speech community on teachers’ interactive metadiscourse strategies. The article concludes with a few implications for metadiscourse research in spoken academic genres.

## Introduction

English for academic purposes (EAP) courses are primary sources of English language teaching in many higher education institutions of the world. Their major function is to assist academically oriented L2 learners to gain literacies and skills necessary to navigate a diverse range of complex academic discourses and be successful participants in the academy (Lee and Subtirelu, 2015). Therefore, classroom comprehension is of paramount significance for EAP students. However, a it poses a threat to non-native English-speaking students due to many factors, such as their insufficient knowledge of English lexicon, failure to appreciate the structural organization of lectures, and lack of pertinent cultural backgrounds (e.g., Chaudron and Richards, 1986; Dunkel and Davis, 1994; Lynch, 2011; Deroey, 2012; Nesi, 2012; Nazari et al., 2020).

Over the past decades, a number of researchers have attempted to explore the various ways of facilitating students’ classroom comprehension and enhancing teachers’ instructional effect (Biber, 2006; Walsh et al., 2011; Riordan, 2018; Nazari and Atai, 2022). Many researchers have noticed the role of metadiscursive resources, including interactive and interactional devices, in classroom teaching. Although Dunkel and Davis (1994) claimed that there is no obvious positive correlation between these discourse markers and lecture comprehension, the majority of studies have suggested that interactive devices, by means of explicitly signaling text structure, have an important effect on both first- and second-language listening comprehension (e.g., Jung, 2003; Jalilifar and Alipour, 2007; Heshemi et al., 2012).

Nevertheless, existing research on metadiscourse has been mainly conducted in a range of written academic genres, such as in research articles (Hong and Cao, 2014; Jiang and Hyland, 2017; Hyland and Jiang, 2018; Li and Xu, 2020), news articles (Makkonen-Craig, 2011; Fu and Hyland, 2014; Peterlin and Moe, 2016), business and commercial genres (Fu, 2012; López-Ferrero and Bach, 2016; Al-Subhi, 2021), and editorials (Khabbazi-Oskouei, 2013, 2016; Shokouhi et al., 2015). By contrast, spoken genres are comparatively understudied, although they have aroused increasing attention. “Speaking has entered the scene much more recently, as in other approaches to academic discourse” (Ädel and Mauranen, 2010, p. 1). To date, very few cross-cultural studies on metadiscourse in spoken academic genres have been made, in particular across the Chinese and British contexts. Such gap of metadiscourse research in spoken academic genres and the crucial role of metadiscursive strategies in facilitating students’ comprehension in classroom teaching settings call for an in-depth study on teachers’ interactive metadiscourse use in classroom teaching settings, in particular in the EAP teaching context. To this end, the current research is based on Hyland’s (2005, 2019) interpersonal model of metadiscourse to make a comparative study of the interactive metadiscourse use by EAP teachers across the British and Chinese contexts.

## Literature review

### The interpersonal model of metadiscourse

Metadiscourse has been conceptualized in either the broad approach or the narrow approach (Ädel and Mauranen, 2010; Hyland and Jiang, 2022). This research follows a broad approach represented mainly by Hyland, as it is aimed at exploring not only teachers’ discourse organization but also their interaction with students in classrooms. Metadiscourse is defined as the cover term for the self-reflective expressions used to negotiate interactional meanings in a text, assisting the writer (or speaker) to express a viewpoint and engage with readers as members of a particular community (Hyland, 2005). In his interpersonal model, Hyland (2005) divided metadiscourse into two broad categories, interactive and interactional metadiscourse, as shown in Table 1. Although Hyland’s (2005) interpersonal model of metadiscourse is mainly designed to investigate written academic discourse, this model has demonstrated its robustness and effectiveness in exploring the discourse organization and audience involvement mechanisms in spoken academic discourse, in particular teachers’ classroom instructional discourse after slight modification of certain metadiscourse markers (e.g., Lee and Subtirelu, 2015; Zhang, 2017).

**TABLE 1:** Hyland’s (2005) interpersonal model of metadiscourse.

Categories	Function	Examples
**Interactive**	**Help to guide the reader through the text**	**Resources**
Transitions	Express relations between main clauses	In addition; but; and
Frame markers	Refer to discourse acts, sequences, or stages	Finally; to conclude; my purpose is
Endophoric markers	Refer to information in other parts of the text	Noted above; see Figure 1 below; in section 2
Evidentials	Refer to information from other texts	According to X; Z states
Code glosses	Elaborate propositional meanings	Namely; e.g., such as; in other words
**Interactional**	**Involve the reader in the text**	**Resources**
Hedges	Withhold commitment and open dialogue	Might; perhaps; possible;
Boosters	Emphasize certainty or close dialogue	In fact; definitely
Attitude markers	Express writer’s attitude to proposition	Unfortunately; I agree
Self-mentions	Explicit reference to author(s)	I; we; my; me; our
Engagement markers	Explicitly build relationship with reader	Consider; note

### Metadiscourse and English for academic purposes courses

In the past decade or so, an increasing number of scholars have noticed the significance of metadiscourse in spoken academic genres (e.g., Zare and Talakoli, 2017; Qiu and Jiang, 2021; Zhang and Lo, 2021; Doiz and Lasagabaster, 2022; Kashiha, 2022; Nazari and Atai, 2022). In particular, some scholars have investigated metadiscourse use in monologic and dialogic types of classroom discourse (Zare and Talakoli, 2017). For example, by adopting Ädel’s (2010) taxonomy of metadiscourse, Zare and Talakoli (2017) compared the functions of personal metadiscourse in academic monologic and dialogic speech, which were represented, respectively, by classroom lectures and discussions. Employing Hyland’s (2005) interpersonal model of metadiscourse, Lee and Subtirelu (2015) made a comparison between teachers’ use of metadiscourse in EAP lessons and academic lectures. These three comparative studies of metadiscourse use in monologic and dialogic types of classrooms share rather similar conclusions. They all show that interactive metadiscourse is used more frequently in monologic speech events, while interactional metadiscourse is more common in dialogic ones due to their respective discourse functions in the two pedagogical contexts. This sheds new light on our understanding of metadiscourse use in classroom contexts. However, very few studies have been conducted in these aspects, and far more research is still needed.

Furthermore, in light of the aforementioned role in assisting lesson comprehension, interactive metadiscourse seems to deserve more scholarly attention in recent years. Indeed, this has drawn increasing scholastic attention in recent years. For instance, based on a corpus of four English medium instruction (EMI) teachers’ interactive metadiscourse use in the Chinese context, Zhang and Lo (2021) examined how different types of interactive metadiscourse expressions are used in university lectures in the science discipline and how these expressions facilitate knowledge construction. They found that transition markers and frame markers were the two most frequently used interactive metadiscourse categories. Within transition markers, the frequently use subcategory marking consequence (e.g., *because* and *so*) indicates that explaining was an important feature of classroom instruction. Frame markers were used as an important means to signal the macro-structure and stage of the lectures. Immediately following this, Doiz and Lasagabaster (2022) investigated four English teachers’ interactive metadiscourse in English classrooms in Spain and made a comparison of their research results with those of Zhang and Lo (2021). They demonstrated the overwhelmingly higher frequency of transition markers and frame markers of EMI teachers in Spain against that in the Chinese context and also pointed out some differences in the linguistic realization of specific metadiscourse markers between EMI teachers across Spain and China.

Taken together, such a crucial role of interactive metadiscourse in classroom comprehension, its specific functions in organizing classroom instruction, and the cross-contexts features make it all the more interesting and worthwhile to explore the intricate nature of English teachers’ interactive metadiscourse in various contexts. Following this line of thought, this study concentrates on the interactive metadiscourse (i.e., transitions and frame markers in the present research) used by native English-speaking EAP writing teachers in the United Kingdom and their non-native counterparts in the Chinese contexts and formulates the following two questions:

(1) How is EAP teachers’ interactive metadiscourse different from and similar to each other across the British and Chinese EAP contexts?
(2) What are the possible reasons for the similarities and variations of interactive metadiscourse use between EAP teachers across the British and Chinese contexts?

## Methodology

### Data collection and corpus compilation

This research is part of a research project in teachers’ classroom metadiscourse use conducted across the United Kingdom and China. Prior to the data collection, ethical approval for this study was obtained from the Research Ethics and Governance Committee of the authors’ institution to ensure the rights of the participants and the integrity, quality, and transparency of the research. Then a questionnaire was delivered to get access to and select native and non-native English-speaking EAP teacher participants, respectively, for this research in both British and Chinese higher education institutions. Teachers and their students were informed fully about the purpose, methods, and intended possible uses of the research, and what their participation in the research entails. Each teacher participant’s classroom teachings, which last for 90 or 120 min, were recorded for two sessions.

These video recordings were then transcribed verbatim to facilitate further analysis. Altogether 16 sessions of eight teachers’ classroom teaching, two sessions by each of the four teachers from the British and the Chinese contexts, respectively, were selected for this study. Finally, two sub-corpora including the classroom discourse of four native English-speaking EAP teachers in the United Kingdom (ET sub-corpus) and four in China (CT sub-corpus) were compiled. To calculate the normalized frequency, each teacher’s classroom talk was restored in a separate file by eliminating students’ talk and the overall information. The total verbatim transcript of their classroom discourse amounts to 70,073 words. By sifting out student talk, the total amount of teacher discourse is 66,035 words. Among them, 32,860 words constitute the teacher discourse in the ET sub-corpus, and 33,175 words in the CT sub-corpus.

### Identification of metadiscourse markers

A corpus-based method was employed in the current research to retrieve potential items of metadiscourse, complemented by a manual analysis of each metadiscourse marker to sift out those irrelevant linguistic items. The concordance tool of AntConc was used to observe the immediate context of each metadiscourse item. In view of the context-dependent nature of metadiscourse markers, each linguistic item was judged by the specific function it performs in its particular context and thus warrants the manual identification of metadiscourse items. Moreover, following Ädel (2010), quoted materials and dysfluencies are excluded from the metadiscourse markers. During this process, the two authors crosscheck part of the identified instances of metadiscourse markers, until a final agreement was achieved concerning the disagreed linguistic items.

The identification of metadiscourse markers also takes into account the findings from Hyland’s (2005, 2019) interpersonal model and some other relevant research. This is due to the aforementioned fact that Hyland’s research of metadiscourse use mainly focuses on written discourse. Due to the differences of linguistic features between written and spoken genres (Ädel, 2010), as mentioned before, there are inevitably some instances of metadiscourse markers specific to spoken discourse but not included in Hyland’s (2005) metadiscourse list. Therefore, the current study also makes reference to reported instances from other metadiscourse research into spoken academic discourse, for example, Lee and Subtirelu’s (2015) research into metadiscourse use by EAP teachers and lecturers.

### Data analysis

**FIGURE 1:**
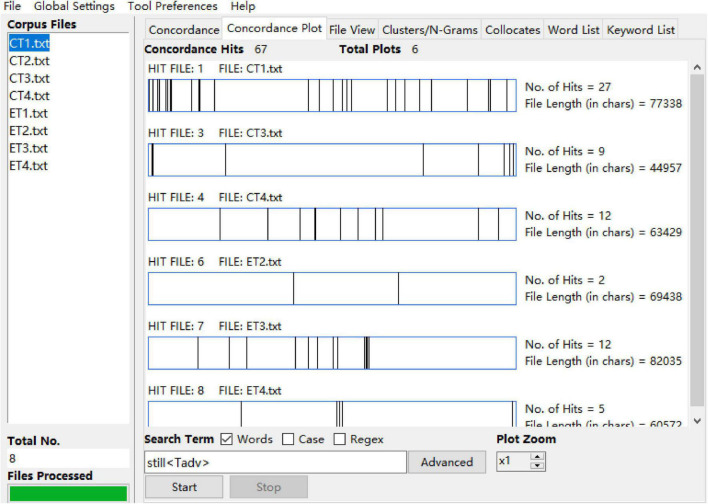
Distribution of metadiscourse marker of *still* in AntConc.

The procedure of data analysis was followed on the basis of pertinent research questions. The frequencies, distributions, and ranges (representing the number of teachers used particular metadiscourse item) of metadiscourse markers used by teachers in the two sub-corpora are the key information needed for this study. Thus, a quantitative discourse analysis was carried out. First, the raw frequencies of certain metadiscourse items can be generated by importing the coded text into AntConc 4.0 and inputting the metadiscourse markers into the search term tool. For example, in Figure 1, the metadiscourse item *still*, together with its coding mark < *Tconj* > , is searched by clicking the “start” button. After that, by clicking the Concordance Plot tool at the upper part of the software, an overall landscape of the total number of instances used by individual teachers of this metadiscourse item is generated. However, the number of total instances generated before is the raw frequency of *still*. Such frequencies are then normalized against per thousand words to generate the normalized frequencies of all the metadiscourse items to make them comparable to each other.

### Analytical framework

Enlightened by Hyland’s (2005, 2019) interpersonal model of metadiscourse, two categories of interactive metadiscourse, transition markers and frame markers, were selected for analysis in this study. The sub-functional categories or pragmatic functions of these two types of metadiscourse markers and their coding examples are shown in Table 2.

**TABLE 2:** Pragmatic functions of interactive metadiscourse markers.

Interactive MD markers	Pragmatic functions	Examples with codes
Transitions	Additions	*And* < *Tconj* >
	Comparisons	*But* < *Tconj* >
	Consequences	*As a result* < *Tconj* >
Frame markers	Sequencing	*First* < *Fnum* >
	Labeling stage	*Overall* < *Fadv* >
	Announcing goal	*Want* < *Fverb* >
	Shifting topic	*So* < *Fconj* >

Transition markers mainly refer to conjunctions or adverbial phrases that assist the addressees to better understand pragmatic connections between steps in an argument. Transitions in the current study consist of three pragmatic functions, namely, additions, comparisons, and consequences. An analysis of data reveals that the linguistic realizations of transitions are generally represented by the lexical category of conjunctions and multi-word expressions. Second, frame markers are generally used to mark text boundaries or elements of the schematic text structure, which can be divided into four pragmatic functions. Specifically, they can be used to sequence parts of a text and act as more explicit additive relations, such as *first*, *then*, and *next*, to explicitly label text stages, such as *to summarize* and *in sum*, to announce discourse goals, as in *my purpose is* and *I want to*, and to signal topic shifts, for example, by using *well*, *right*, and *now*.

## Results

### Transitions

#### Additions

The additive function of transition markers generally serves to add some elements to an argument. It is mainly composed of conjunctions and adverbial phrases that help the addressees follow and interpret pragmatic relations between steps in an argument. Table 3 presents the frequencies, types, and ranges of additive markers in the two sub-corpora. In relation to frequencies, teachers in the ET sub-corpus employ additive metadiscourse 15.64 times per thousand words, while those in the CT sub-corpus use additions with 15.43 instances per thousand words. The log-likelihood value (0.05) indicates that there is no statistically significant difference in the use of additions across the two sub-corpora. As for the types of lexical items, teachers in the ET sub-corpus adopt six types, while those in the CT sub-corpus use eight types. In terms of the ranges of these lexical items, they are characterized by the pervasive use of *and also* by each of the individual teachers in the two sub-corpora to introduce an additive proposition. In effect, previous research noted that *and* is so prevalent that it is sometimes left out from rhetorical analysis, being regarded as the default option of “marking conjunctive relations” of addition (Hyland and Jiang, 2018, p. 21).

**TABLE 3:** Summary of additive transitions in both sub-corpora.

No.	Lexical items	ETs			CTs			Log-likelihood value*
				
		RawFrq.	NmlFrq.	Range	RawFrq.	NmlFrq.	Range	
1	And	440	13.39	4	331	9.98	4	16.52****
2	Also	26	0.79	4	111	3.35	4	−55.98****
3	Still	19	0.58	3	48	1.45	3	−12.70***
4	Again	26	0.79	4	5	0.15	2	15.78****
5	Further	2	0.06	1	11	0.33	3	−6.77**
6	Equally	1	0.03	1	2	0.06	1	–0.33
7	At the same time	0	0.00	0	2	0.06	1	–2.75
8	Besides	0	0.00	0	2	0.06	1	–2.75
	**Total**	**514**	**15.64**		**512**	**15.43**		**0.05**

*p < 0.05; **p < 0.01; ***p < 0.001; ****p < 0.0001.

#### Comparisons

Comparative transitions are used with the least frequency among the three transitional devices. They refer to arguments or ideas which are similar to or different from each other. However, no metadiscoursal instance of similarity is observed throughout the dataset. Some instances such as *similarly*, *likewise*, and *in the same way* are all quoted examples from the informants during the exercise task of classroom instruction. Therefore, comparisons as transition metadiscourse markers in the current research mainly refer to contrastive relations. Table 4 demonstrates the frequencies, types, and ranges of comparisons used in the two sub-corpora. With regard to frequencies, teachers in the ET sub-corpus employ significantly more comparisons than their counterparts in the CT sub-corpus at *p* < 0.0001. As for lexical types, teachers in the ET sub-corpus employ altogether 11 types of lexical items, obviously more than those in the CT sub-corpus (three types). This exhibits a slightly different pattern from that of additions, for which teachers in the CT sub-corpus use two types more than those in the ET sub-corpus. In terms of ranges, it is evident that the lexical item *but* appears in each of the eight teachers’ classroom discourses across the two sub-corpora. All the other lexical items in either sub-corpus appear in one or two teachers’ discourse. This indicates the pervasive use of *but* as a comparative transition among other alternative lexical items. Resembling the use of *and* as the default option of “marking conjunctive relations” of addition (Hyland and Jiang, 2018, p. 21), *but* might be regarded as the default option for expressing comparative and consequential relations in academic speech contexts.

**TABLE 4:** Summary of comparative transitions in both sub-corpora.

No.	Lexical items	ETs			CTs			Log-likelihood value*
				
		RawFrq	NmlFrq	Range	RawFrq	NmlFrq	Range	
1	But	200	6.09	4	98	2.95	4	36.61****
2	Rather than	7	0.21	2	0	0	0	9.77**
3	Yet	5	0.15	2	0	0	0	6.98**
4	Even if	4	0.12	2	0	0	0	5.58*
5	Although	1	0.03	1	2	0.06	1	–0.33
6	Even though	3	0.09	2	0	0	0	4.19*
7	However	2	0.06	1	1	0.03	1	0.35
8	Whereas	3	0.09	2	0	0	0	4.19*
9	On the other hand	2	0.06	1	0	0	0	2.79
10	Though	2	0.06	2	0	0	0	2.79
11	While	2	0.06	2	0	0	0	2.79
	**Total**	**231**	**7.03**		**101**	**3.04**		53.54****

*p < 0.05; **p < 0.01; ****p < 0.0001.

#### Consequences

Consequential transitions mark that a conclusion is being drawn or justified, or that an argument is being rejected. Analysis showed that teachers in the ET sub-corpus use consequences with the highest frequency among the three transitional devices they use. On the other hand, teachers in the CT sub-corpus employ consequences with the second highest frequency. Table 5 presents the frequencies, types, and ranges of consequential lexical items in both sub-corpora. Concerning frequencies, teachers in the ET sub-corpus make significantly more frequent use of consequential transitions than those in the CT sub-corpus at the *p* < 0.0001. With regard to lexical types, distinct from their discrepancies in additions and comparisons, both groups of teachers use consequences with six types of lexical items. In relation to ranges, two lexical items *so* and *because* are used extensively by each of the teachers in both sub-corpora. In particular, *so* is used with predominantly higher frequencies as opposed to other lexical items in both sub-corpora. This may prove that *so* may be deemed as the default form to convey consequential relations between propositions.

**TABLE 5:** Summary of consequential transitions in both sub-corpora.

No.	Lexical items	ETs			CTs			Log-likelihood value*
				
		RawFrq.	NmlFrq.	Range	RawFrq.	NmlFrq.	Range	
1	So	495	15.06	4	209	6.30	4	122.36****
2	Because	129	3.93	4	81	2.44	4	11.53***
3	So that	4	0.12	3	17	0.51	3	−8.54**
4	Since	1	0.03	1	8	0.24	3	−6.13*
5	Therefore	2	0.06	1	3	0.09	1	–0.19
6	Thus	0	0	0	4	0.12	2	−5.51*
7	As a result	1	0.03	1	0	0	0	1.4
	**Total**	**632**	**19.23**		**322**	**9.71**		105.57****

*p < 0.05; **p < 0.01; ***p < 0.001; ****p < 0.0001.

### Frame markers

#### Sequencing

Sequencing refers to the order of different parts of a discourse or an argument, usually serving as explicit additive relations. It is the second most frequently used frame marker following the shifting topic category. Table 6 reveals the frequencies, types, and ranges of individual lexical items used by teachers in the two sub-corpora. First, it reveals that teachers in the ET sub-corpus use sequencing devices less frequently than those in the CT sub-corpus. Specifically, the log-likelihood value indicates that teachers in the ET sub-corpus use significantly less sequencing devices than their counterparts in the CT sub-corpus at *p* < 0.0001. Second, it can be noticed that both groups of teachers use quite similar numbers of lexical types. Teachers in the ET sub-corpus use 10 types of lexical items, while those in the CT sub-corpus use 11. Third, in terms of the ranges of lexical items such sequencing markers including *then*, *first*, *second*, *last*, and *next* are used extensively in both sub-corpora. The high frequency of sequencing markers in the CT sub-corpus is largely due to the larger proportion of these five lexical items. In addition, teachers in the CT sub-corpus also use *third* and *first of all* in considerably high proportions. This evidences previous research that tertiary-level classroom instructions are heavily signposted (Swales, 2001).

**TABLE 6:** Summary of sequencing in both sub-corpora.

No.	Lexical items	ETs			CTs			Log-likelihood value*
				
		RawFrq.	NmlFrq.	Range	RawFrq.	NmlFrq.	Range	
1	Then	163	4.96	4	228	6.87	4	−10.24**
2	First	60	1.83	4	153	4.61	4	−41.12****
3	Second	36	1.10	4	84	2.53	4	−19.29****
4	Last	32	0.97	4	51	1.54	4	−4.21*
5	Next	36	1.10	4	24	0.72	4	2.53
6	Start (s/ing)	22	0.67	4	13	0.39	2	2.43
7	Third	2	0.06	1	29	0.87	4	−27.89****
8	First of all	2	0.06	2	11	0.33	4	−6.77**
9	Begin	2	0.06	1	4	0.12	3	–0.66
10	Firstly	1	0.03	1	3	0.09	1	–1.03
11	Secondly	0	0.00	0	1	0.03	1	–1.38
	**Total**	**356**	**10.83**		**601**	**18.12**		−61.11****

*p < 0.05; **p < 0.01; ****p < 0.0001.

Moreover, the relatively high frequency of *first* also resonates with the finding of Yang (2014), who notes that *first/first of all* often acts as an important navigational aid for the students to “locate learning in time and space” (Walsh, 2011, p. 208). This is also supported by teachers’ varying strategies of initiating a topic, including the use of *start (s/ing)*, *begin, firstly*, and *first of all*, which together would account for 2.65 and 5.55 instances per thousand words, respectively, in the ET and CT sub-corpora. However, the current research diverges from that of Yang (2014) in that the overall frequency of these initial sequence markers is still lower than that of *then*. This may demonstrate that there are greater requirements for every following step of the sequences. Furthermore, the thorough analysis of the dataset shows that there are large proportions of teacher monolog in the CT sub-corpus. These successive sequencing lexical items also function as cohesive devices (Halliday and Hasan, 1976) in organizing classroom discourse.

#### Labeling stages

As noted above, labeling stages is the least frequently used device among the four pragmatic functional categories realizing frame markers. Table 7 provides an overview of the frequencies, types, and ranges of lexical items used by teachers in the ET and CT sub-corpora, respectively. First, the frequency analysis reveals that teachers in the CT sub-corpus use labeling-stage frame markers at a slightly higher frequency (0.39 ptw) than those in the ET sub-corpus (0.27 ptw). The log-likelihood value further shows that there is no significant difference between their frequencies in using labeling-stage frame markers. Second, in terms of lexical types, similar to the aforementioned sequencing category, teachers in the ET sub-corpus employ one type less than those in the CT sub-corpus. Specifically, teachers in the ET sub-corpus use five types of labeling-stage markers, while those in the CT sub-corpus employ six types. Third, regarding ranges, there are no shared lexical items widely used by either or both of the two groups of teachers. This evidences the research of Yan (2010) in that labeling-stage frame markers may not be a characteristic of spoken language, in particular face-to-face communication.

**TABLE 7:** Summary of labeling stages in both sub-corpora.

No.	Lexical items	ETs			CTs			Log-likelihood value*
				
		RawFrq.	NmlFrq.	Range	RawFrq.	NmlFrq.	Range	
1	Conclude	0	0.00	0	5	0.15	2	−6.88**
2	At this point	3	0.09	2	0	0.00	0	4.19*
3	Summarize	0	0.00	0	3	0.09	1	−4.13*
4	By far	2	0.06	2	0	0.00	0	2.79
5	For the Moment	2	0.06	2	0	0.00	0	2.79
6	Sum up	0	0.00	0	2	0.06	2	–2.75
7	All in all	0	0.00	0	1	0.03	1	–1.38
8	In short	1	0.03	1	0	0.00	0	1.40
9	In sum	1	0.03	1	0	0.00	0	1.40
10	Restate	0	0.00	0	1	0.03	1	–1.38
11	Review	0	0.00	0	1	0.03	1	1.38
	**Total**	**9**	**0.27**		**13**	**0.39**		–0.69

*p < 0.05; **p < 0.01.

#### Announcing goals

Following shifting topics and sequencing, announcing goals rank third among the four pragmatic functions realizing frame markers. Table 8 demonstrates the frequencies, types, and ranges of the lexical items employed by teachers in the ET and CT sub-corpora. In the first place, teachers in the ET sub-corpus use announcing goals with 3.38 instances per thousand words, which is slightly higher than those in the CT sub-corpus. The log-likelihood value (0.81) indicates that there is no significant difference in the use of announcing goals between teachers in the two sub-corpora. Second, teachers in the ET sub-corpus use five types of lexical items, one type more than those used by teachers in the CT sub-corpus. Specifically, the lexical item *aim*, which is used twice by one teacher in the ET sub-corpus, has no instance in the CT sub-corpus. This may indicate that the difference in using this lexical item could be due to the particular characteristic of individual teachers, but not a pervasive phenomenon. Third, compared with other lexical items, the expression *want to* is used widely and most frequently by every teacher across both sub-corpora. This reveals that *want to* is the most commonly used expression for teachers announcing goals in the classroom teaching process.

**TABLE 8:** Summary of announcing goals in both sub-corpora.

No.	Lexical items	ETs			CTs			Log-likelihood value*
				
		RawFrq.	NmlFrq.	Range	RawFrq.	NmlFrq.	Range	
1	Want to	50	1.52	4	47	1.42	4	0.12
2	Focus	35	1.07	3	22	0.66	3	3.12
3	Purpose(s)	16	0.49	4	21	0.63	2	–0.63
4	Would like to	8	0.24	4	9	0.27	3	–0.05
5	Aim	2	0.06	1	0	0.00	0	2.79
	**Total**	**111**	**3.38**		**99**	**2.98**		**0.81**

*p < 0.05.

#### Shifting topics

Among the four pragmatic functions of frame markers, shifting topics is the most frequently used category. The frequencies, types, and ranges of linguistic expressions used by teachers in the ET and CT sub-corpora can be demonstrated in Table 9. First, teachers in the ET sub-corpus use shifting topic markers with 20.97 instances per thousand words, less than those used by teachers in the CT sub-corpus (23.24 ptw). Moreover, the log-likelihood values indicate that teachers in the ET sub-corpus use significantly less shifting topic markers than those in the CT sub-corpus at *p* < 0.05. Second, teachers in the ET sub-corpus use nine types of lexical items, compared with eight types used by those in the CT sub-corpus. One lexical item *well*, which is widely used by every teacher in the ET sub-corpus, does not occur in the CT sub-corpus. This might reflect one of the distinctive characteristics of metadiscourse use between the two groups of teachers. Third, in terms of the ranges of lexical items, *okay*, *so*, and *now* are three widely used lexical items by every teacher in both ET and CT sub-corpora. They may represent the common features of teachers’ classroom discourse by both native and non-native EAP teachers and in both Chinese and British educational settings. In addition, compared with their sporadic occurrences in the CT sub-corpus, other lexical categories, such as *right*, *well*, *back to*, and *move on*, are also used extensively by each teacher in the ET sub-corpus.

**TABLE 9:** Summary of shifting topics in both sub-corpora.

No.	Lexical items	ETs			CTs			Log-likelihood value*
				
		RawFrq.	NmlFrq.	Range	RawFrq.	NmlFrq.	Range	
1	Okay	293	8.92	4	514	15.49	4	−59.21****
2	So	191	5.81	4	171	5.15	4	1.3
3	Now	41	1.25	4	57	1.72	4	–2.47
4	Right	53	1.61	4	10	0.30	3	32.62****
5	Well	52	1.58	4	0	0.00	0	72.58****
6	All right	24	0.73	3	7	0.21	1	10.02**
7	Back to	22	0.67	4	3	0.09	1	16.49****
8	Move on	10	0.30	4	4	0.12	1	2.71
9	Move	3	0.09	3	5	0.15	1	–0.49
	**Total**	**689**	**20.97**		**771**	**23.24**		−3.86*

*p < 0.05; **p < 0.01; ****p < 0.0001.

## Discussion

### Similarities of metadiscourse use across the ET and CT sub-corpora

This section discusses the considerable similarities in metadiscourse use between teachers in the ET and CT sub-corpora, in the sense of both individual metadiscourse categories and individual lexical items within each metadiscourse category.

In relation to individual metadiscourse categories, teachers in both sub-corpora use transitions and frame markers with comparatively higher frequencies than endophoric markers and code glosses. This finding is in line with Yan (2010) and Lee and Subtirelu (2015). It also evinces that teachers in both sub-corpora attach considerable attention to organizing and guiding students through the classroom discourse at both local (realized by transition markers) and global (realized by frame markers) levels (Chaudron and Richards, 1986; DeCarrico and Nattinger, 1988; Zhang, 2017). Both transition markers, such as *and* or *but*, and frame markers, such as *first* or *so*, may contribute to a coherent classroom discourse and help signal shifts in discourse trajectories (Crawford Camiciottoli, 2005). Since students in both contexts of the current study are at the stage of learning language skills, teachers in both sub-corpora are sensitive to students’ needs for assistance in navigating through the instructional process. Teachers’ use of such interactive markers may serve as signposts to help relieve the cognitive burden on the part of students in processing the classroom instruction that normally lasts for approximately an hour (Cazden, 2001; Bu, 2014). As such, students would benefit from the effort made by teachers in achieving optimal relevance with minimal processing effort in the interpretation of academic information.

In terms of individual lexical items within each metadiscourse category, the first aspect concerns that both groups of teachers make frequent use of transition markers such as *and*, *but*, and *so*, respectively, to display the additive, comparative, and consequential transitions between discourse segments. In effect, previous research also found that *and* is so prevalent that it is sometimes left out from rhetorical analysis because it is regarded as the default option of “marking conjunctive relations” of addition (Hyland and Jiang, 2018, p. 21). The current research may develop this view and demonstrate that *but* and *so* likewise are so prevalent that they may also be deemed as the default options of expressing comparative and consequential relations in academic speech contexts.

Second, teachers in both sub-corpora use the frame markers *then*, *first*, and *second* with considerably high frequencies to indicate sequencing relations. These sequencing frame markers can serve as an important navigational aid for the students to “locate learning in time and space” (Walsh, 2011, p. 208). This confirms Fung and Carter’s (2007) view that discourse markers like *firstly, secondly*, and *then* are used frequently in teachers’ classroom discourse to signal and segment the logical sequence. However, the current research reports exceptionally higher frequencies of *then* compared with *first* and *second*, which seems to be inconsistent with Yang’s (2014) finding that *first* is predominantly widespread in Chinese teachers’ classroom discourse, sometimes without using subsequent logical connectors like *secondly* and *thirdly*. Nevertheless, this might be compensated for by other forms of expressions with a similar meaning to *first*, either in freestanding forms such as *firstly*, *start*, and *begin*, or in the multiword expression *first of all* in the current research.

The third feature shared by teachers in ET and CT sub-corpora is the extensive use of framing markers *okay*, *so*, and *now*. These are the top three frequently used lexical items by every teacher in both ET and CT sub-corpora. This is partly in conformity with previous studies (Sacks et al., 1974; Schiffrin, 1987) which found that *okay* and/or *so* are common pre-closing devices to open another round of talk prior to conversational closure. Moreover, Carter and McCarthy (2006) also noted that these frame markers marking shifting topics are commonly used at the opening/closing positions of a topic. They can perform both interactive and social function at the same time in classroom discourse (Walsh, 2006; Fung and Carter, 2007). This enriches previous studies on discourse markers (e.g., Hellermann and Vergun, 2007; Evison, 2009). These signposting devices can function as a lubricant in teacher–student interaction to reduce understanding difficulties, incoherence, and social distance between teachers and students (Yang, 2014).

### Differences of metadiscourse use across the ET and CT sub-corpora

The current research has sought to make an in-depth analysis of two other aspects of variations in metadiscourse use between teachers in the two sub-corpora. The first difference relates to the use of some metadiscourse markers with different functions. The second aspect concerns salient metadiscourse devices which may occur with strikingly high frequencies in one sub-corpus but with very few or no instances in another.

The first difference of transitions and frame markers across teachers in the ET and CT sub-corpora is the use of consequence transition marker *so* in the ET sub-corpus as opposed to the frame markers *first* and *then* in the CT sub-corpus. As mentioned earlier, teachers endeavor to construct a coherent classroom discourse at both the local (realized by transition markers) and global (realized by fame markers) levels. However, results from the current research reveal that the teachers in the ET sub-corpus are more inclined to organize classroom discourse at the local level typically by virtue of consequential relationships. This is realized by the significantly high frequencies of the transition marker *so*. Yet the case is opposite on the part of teachers in the CT sub-corpus, who mainly frame the sequence of the discourse by the frame markers *first* and *then*. This may extend previous research on teachers’ classroom metadiscourse use (e.g., Lee and Subtirelu, 2015; Zhang, 2017) by demonstrating that Chinese EAP teachers and those in the United Kingdom show variations in their logical preferences when delivering classroom instruction. Admittedly, even though transitions and frame markers are employed with varying frequencies in the two sub-corpora, these two categories constitute the major interactive devices.

With regard to the second aspect, an obvious discrepancy concerns the frame marker *well*, which is used as a shifting topic device. It occurs with a relatively high frequency in the ET sub-corpus, however, with no instance in the CT sub-corpus. Such a discrepancy may be explained by the development order of acquisition (Hays, 1992; Cf. Hellermann and Vergun, 2007). In a study on the use of different types of discourse markers by Japanese learners of English in their first, second, or third year of study, Hays (1992) found that while discourse markers *but*, *and*, and *so* are used frequently, very few learners use *well*. This led him to speculate that there might exist a developmental order for the acquisition of discourse markers. Discourse markers that are on the ideational plane have greater semantic weight and are taught and used first, whereas those that are more purely pragmatic, interactional discourse markers appear later in the subjects’ speech. This speculation is supported by Trillo’s (2002) corpus-based study that compares the use of discourse marker usage between native speakers and L2 learners of English and also finds that learners of English use the discourse markers *well*, among others, with a much lower frequency than native speakers.

### Impact of speech community and discourse community on teachers’ rhetorical strategies

The aforementioned two sections illustrated the similarities and differences of metadiscourse use between teachers in the ET and CT sub-corpora and explored their possible reasons, respectively. Taken together, however, this vast similarities and differences of teachers’ rhetorical characteristics may be explained under the constructs of discourse community and speech community.

Based on Swales (1990), a discourse community generally has a broadly agreed set of common goals, and its members share a suitable degree of content and discursive expertise. Following this thought, it is arguable that the EAP teachers in both ET and CT sub-corpora can be regarded as belonging to a specific type of EAP teaching discourse community. Members, such as teachers, in one discourse community may demonstrate more or less similar patterns of behavior in their classroom discourse in order to sustain their professional membership, such as the use of metadiscourse markers to help students navigate through the lesson. This may lead to extensive similarities in their metadiscourse use in classroom teaching.

Specifically, this research demonstrated considerable similarities in interactive metadiscourse use across the two groups of teachers, in the sense of both individual metadiscourse categories and individual lexical items within each metadiscourse category. Such similar features may be due to the fact that teachers’ classroom discourse in both sub-corpora, although located in difference cultural and educational settings, falls into the same discourse community. That is to say, they both belong to spoken academic discourse or, more specifically, classroom teaching discourse. This is also corroborated by the fact that these both of two groups of teachers are delivering EAP writing courses, which are specifically selected for the present study to reduce the effects of other factors apart from cultural and educational settings. Previous research (Swales, 1990; Arminen, 2005; Abdi et al., 2010; Lee, 2016) has noticed that specific genres can restrict the discourse conventions of communication. Thus, it might be concluded that the norms and conventions of the classroom teaching discourse genre constrain the metadiscourse use of these teachers, irrespective of their variant cultural and educational backgrounds.

Meanwhile, discourse community is often contrasted with a speech community, which is defined as “a group of people who naturally share a language (e.g., native speaker of English) in terms of grammar, lexicon, etc.” (Abdi et al., 2010, p. 1670). In other words, a speech community refers to a group of people whose membership is naturally formed due to factors such as geographical locations and largely cannot be chosen. Specifically, this research also demonstrated considerable discrepancies in the metadiscourse use between teachers in the ET and CT sub-corpora in terms of both some metadiscourse markers with variant or roughly contrastive functions, and some salient metadiscourse devices which may occur with markedly high frequencies in one sub-corpus but with very few or no instances in another. In addition to the aforementioned possible reasons such as logical preferences and development order of acquisition, another more general reason may be that teachers in the two sub-corpora belong to two different speech communities.

As noted by Abdi et al. (2010), people may fall into different speech communities due to their geographical locations and cultural backgrounds, which cause them to share a language and cultural norm that differ from another group of people. Among other factors, the former group of teachers belongs to a part of the Western world in which English is spoken as the first language, whereas the latter belongs to the eastern world in which English is learned and spoken as a foreign language. Such a discrepancy in the speech community may result in concomitant distinctive features to differentiate them from each other. Moreover, previous research (Swales, 1990; Abdi et al., 2010; Lee, 2016) argues that specific genres have a bearing on discourse use, the present research may further demonstrate that variation in speech communities can also lead to discrepancies in patterns of communication.

## Conclusion

The study investigated the interactive metadiscourse use by EAP teachers across the British and Chinese contexts. It corroborates previous research that teachers attach much importance to organizing and guiding students through the classroom discourse at both local (realized by transition markers) and global (realized by frame markers) levels (Chaudron and Richards, 1986; DeCarrico and Nattinger, 1988; Zhang, 2017). Moreover, considerable alignments and discrepancies of interactive metadiscourse use were observed between EAP teachers in the two cultural and educational settings. The alignments were evidenced in both individual metadiscourse categories and individual lexical items within each metadiscourse category, whereas the discrepancies reside in the different uses of some metadiscourse markers with varying functions, or some salient metadiscourse devices which may occur at strikingly high frequencies in one sub-corpus but with very few or no instances in another. Potential reasons such as variation in logical preferences, development order of acquisition, and the notions of discourse community and speech community were also discussed to illustrate these similarities and differences of metadiscourse use by EAP teachers in the British and Chinese contexts. In addition, other factors, such as individual teachers’ beliefs about EAP language teaching (Basturkmen, 2012), their coping strategies (Nazari and Atai, 2022), pedagogical knowledge (Shulman, 1986), and language awareness (Andrew, 2001, 2007), may also, to some extent, affect teachers’ discursive practices, which are interesting topics but are beyond the scope of the current research due to space limitations.

Admittedly, the research is not without any limitations. First, following the data sampling size of Zhang and Lo (2021) and Doiz and Lasagabaster (2022), this research also selected four teachers in each side for comparison. Such a small corpus may inevitably result in a lack of rigor in the generalizability of the findings in the current research to the broader native and non-native EAP teachers in the United Kingdom and China, or the transferability of those findings to other contexts. Also, the metadiscourse items identified in this study are by no means exhaustive, but merely representative of the current research. They should also be reconsidered according to specific contexts being investigated in further research. Bearing this in mind, the current research is not intended to be generalizable or transferrable but to be explanatory and illustrative of teachers’ classroom discourse in EAP writing courses. Future research may rely on some large-scale corpus to probe into the more generalizable features of teachers’ metadiscourse use between teachers in different cultural contexts. In addition, more varied sources of data could be used to triangulate the findings of the current research, such as using stimulated recall or semi-structured interviews, reflective journals, and questionnaires to investigate teachers’ and students’ perceptions and views of metadiscourse use in classrooms. Having said that, the study presented here has made a useful comparison between the findings of this research and those of existing studies into spoken and written academic genres. Such a discussion situates the results of the current research into a broader academic context and builds up our understanding of teachers’ metadiscourse use in classrooms.

## Data availability statement

The original contributions presented in this study are included in the article/supplementary material, further inquiries can be directed to the corresponding author.

## Ethics statement

The studies involving human participants were reviewed and approved by the Research Ethics and Governance Committee of the College of Arts and Social Sciences at the University of Aberdeen. The patients/participants provided their written informed consent to participate in this study.

## Author contributions

XW conceptualized the study, collected the data, analyzed the data, and wrote the manuscript. HY conceptualized the study, analyzed the data, and reviewed the writing. Both authors listed have made a substantial, direct, and intellectual contribution to the work, and approved it for publication.

## References

[B1] Abdi R. Rizi M. T. Tavakoli M. (2010). The cooperative principle in discourse communities and genres: a framework for the use of metadiscourse. *J. Pragmat.* 42 1669–1679. 10.1016/j.pragma.2009.11.001

[B2] Ädel A. (2006). *Metadiscourse in L1 and L2 English.* Amsterdam: John Benjamins. 10.1075/scl.234

[B3] Ädel A. (2010). Just to give you kind of a map of where we are going: a taxonomy of metadiscourse in spoken and written academic English. *Nordic J. English Stud.* 9 69–97. 10.35360/njes.218

[B4] Ädel A. Mauranen A. (2010). Metadiscourse: diverse and divided perspectives. *Nordic J. English Stud.* 9 1–11. 10.35360/njes.215

[B5] Al-Subhi A. S. (2021). Metadiscourse in online advertising: exploring linguistic and visual metadiscourse in social media advertisements. *J. Pragmat.* 187 24–40. 10.1016/j.pragma.2021.10.027

[B6] Andrew S. (2001). The language awareness of the L2 teacher: its impact upon pedagogical practice. *Lang. Awareness* 13 75–90. 10.1080/09658410108667027

[B7] Andrew S. (2007). *Teacher Language Awareness.* Surrey, UK: Cambridge university press. 10.1017/CBO9780511497643

[B8] Arminen I. (2005). *Institutional Interaction: Studies of Talk At Work.* Surrey, UK: Ashgate Publishing.

[B9] Basturkmen H. (2012). Review of research into the correspondence between language teachers’ stated beliefs and practices. *System* 40 282–295. 10.1016/j.system.2012.05.001

[B10] Biber D. (2006). *University Language: A Corpus-Based Study Of Spoken And Written Registers.* Amsterdam, Netherlands: John Benjamins. 10.1075/scl.23

[B11] Bu J. (2014). Towards a pragmatic analysis of metadiscourse in academic lectures: from relevance to adaptation. *Dis. Stud.* 16 449–472. 10.1177/1461445613519019

[B12] Carter R. McCarthy M. (2006). *Cambridge Grammar of English: A Comprehensive Guide: Spoken and Written English Grammar and Usage.* Cambridge: Cambridge University Press.

[B13] Cazden C. B. (2001). *Classroom Discourse: The Language of Teaching and Learning.* Ports- mouth, NH: Heinemann.

[B14] Chaudron C. Richards J. (1986). The effect of discourse markers on the comprehension of lectures. *Appl. Ling.* 7 113–127. 10.1093/applin/7.2.113

[B15] Crawford Camiciottoli B. (2005). Adjusting a business lecture for an international audience: a case study. *Engl. Specif. Purp.* 24, 183–199. 10.1016/j.esp.2004.05.002

[B16] DeCarrico J. Nattinger J. R. (1988). Lexical phrases for the comprehension of academic lectures. *Eng. Specific Purposes* 7 91–102. 10.1016/0889-4906(88)90027-0

[B17] Deroey K. (2012). What they highlight is: the discourse functions of basic wh-clefts in lectures. *J. Eng. Acad. Purposes* 11 112–124. 10.1016/j.jeap.2011.10.002

[B18] Doiz A. Lasagabaster D. (2022). Looking into english-medium instruction teachers’ metadiscourse: an ELF perspective. *System* 105:102730. 10.1016/j.system.2022.102730

[B19] Dunkel P. A. Davis J. N. (1994). “The effects of rhetorical signaling cues on the recall of English lecture information by speakers of English as a native or second language,” in *Academic Listening: Research Perspectives*, ed. Flowerdew J. (Cambridge: Cambridge University Press.), 55–74. 10.1017/CBO9781139524612.007

[B20] Evison J. M. (2009). “Academic discourse,” in *The Pragmatics Encyclopaedia*, ed. Cummings L. (Routledge), 27–29.

[B21] Fu X. (2012). The use of interactional metadiscourse in job postings. *Dis. Stud.* 14 399–417. 10.1177/1461445612450373

[B22] Fu X. Hyland K. (2014). Interaction in two journalistic genres: a study of interactional metadiscourse. *English Text Construct.* 7 122–144. 10.1075/etc.7.1.05fu 33486653

[B23] Fung L. Carter R. (2007). Discourse markers and spoken English: native and learner use in pedagogic settings. *Appl. Ling.* 28 410–439. 10.1093/applin/amm030

[B24] Halliday M. A. K. Hasan R. (1976). *Cohesion in English.* London: Longman.

[B25] Hays P. R. (1992). Discourse markers and L2 acquisition. *Papers Appl. Ling. Mich.* 7 24–34. 10.1075/ijcl.16.2.04aij 33486653

[B26] Hellermann J. Vergun A. (2007). Language which is not taught: the discourse marker use of beginning adult learners of English. *J. Pragmat.* 39 157–179. 10.1016/j.pragma.2006.04.008

[B27] Heshemi R. Mohammad S. Khodabakhshzade H. Shirvan M. E. (2012). The effect of metadiscourse on EFL learners’ listening comprehension. *J. Lang. Teach. Res.* 3 452–457. 10.4304/jltr.3.3.452-457

[B28] Hong H. Cao F. (2014). Interactional metadiscourse in young EFL learner writing: a corpus-based study. *Int. J. Corpus Ling.* 19 201–224. 10.1075/ijcl.19.2.03hon 33486653

[B29] Hyland K. (2005). *Metadiscourse: Exploring Interaction in Writing.* London: Continuum.

[B30] Hyland K. (2019). *Metadiscourse: Exploring Interaction in Writing*, 2nd Edn. London: Bloomsbury Academic.

[B31] Hyland K. Jiang F. (2018). In this paper we suggest’: changing patterns of disciplinary metadiscourse. *English Specific Purposes* 51 18–30. 10.1016/j.esp.2018.02.001

[B32] Hyland K. Jiang F. (2022). Metadiscourse across languages and genres: an overview. *Lingua* 265:103205. 10.1016/j.lingua.2021.103205

[B33] Jalilifar A. Alipour M. (2007). How explicit instruction makes a difference: metadiscourse markers and EFL learners’ reading comprehension skill. *J. College Read. Learn.* 38 35–52. 10.1080/10790195.2007.10850203

[B34] Jiang F. Hyland K. (2017). Metadiscursive nouns: interaction and cohesion in abstract moves. *English Specific Purposes* 46 1–14. 10.1016/j.esp.2016.11.001

[B35] Jung S. (2003). The effects of organization markers on ESL learners’ text understanding. *Tesol. Quart.* 37 749–759. 10.2307/3588223

[B36] Kashiha H. (2022). Academic lectures versus political speeches: metadiscourse functions affected by the role of the audience. *J. Pragmat.* 190 60–72. 10.1016/j.pragma.2022.01.003

[B37] Khabbazi-Oskouei L. (2013). Propositional or non-propositional, that is the question: a new approach to analysing ‘interpersonal metadiscourse’ in editorials. *J. Pragmat.* 47 93–107. 10.1016/j.pragma.2012.12.003

[B38] Khabbazi-Oskouei L. (2016). Orality in persian argumentative discourse: a case study of editorials. *Iran. Stud.* 49 677–691. 10.1080/00210862.2015.1026250

[B39] Lee J. (2016). ‘There’s intentionality behind it.’: a genre analysis of EAP classroom lessons. *J. Eng. Acad. Purposes* 23 99–112. 10.1016/j.jeap.2015.12.007

[B40] Lee J. Subtirelu N. (2015). Metadiscourse in the classroom: a comparative analysis of EAP lessons and university lectures. *Eng. Specific Purposes* 37 52–62. 10.1016/j.esp.2014.06.005

[B41] Li Z. Xu J. (2020). Reflexive metadiscourse in Chinese and English sociology research article introductions and discussions. *J. Pragmat.* 159 47–59. 10.1016/j.pragma.2020.02.003

[B42] López-Ferrero C. Bach C. (2016). Discourse analysis of statements of purpose: connecting academic and professional genres. *Dis. Stud.* 18 286–310. 10.1177/1461445616634553

[B43] Lynch T. (2011). Academic listening in the 21st century: reviewing a decade of research. *J. Eng. Acad. Purposes* 10 79–88. 10.1016/j.jeap.2011.03.001

[B44] Makkonen-Craig H. (2011). Connecting with the reader: participant-oriented metadiscourse in newspaper texts. *Text Talk* 31 683–704. 10.1515/text.2011.033

[B45] Nazari O. Atai M. R. (2022). An exploratory study of EAP teachers. *Coping Strat.* 106:102764. 10.1016/j.system.2022.102764

[B46] Nazari O. Atai M. R. Birjandi P. (2020). An investigation into iranian EAP teachers’ burnout and its variations in relation to their demographic and organizational characteristics. *Issues Lang. Teach.* 9 93–116.

[B47] Nesi H. (2012). Laughter in university lectures. *J. Eng. Acad. Purposes* 11 79–89. 10.1016/j.jeap.2011.12.003

[B48] Peterlin A. P. Moe M. Z. (2016). Translating hedging devices in news discourse. *J. Pragmat.* 102 1–12. 10.1016/j.pragma.2016.06.009

[B49] Qiu X. Jiang F. (2021). Stance and engagement in 3MT presentations: how students communicate disciplinary knowledge to a wide audience. *J. Eng. Acad. Purposes* 51:100976. 10.1016/j.jeap.2021.100976

[B50] Riordan E. (2018). *). Language for Teaching Purposes: Bilingual Classroom Discourse and the Non-Native Speaker Language Teacher.* Switzerland: Palgrave Macmillan. 10.1007/978-3-319-71005-1_6

[B51] Sacks H. Schegloff E. A. Jefferson G. (1974). A simplest systematics for the organization of turn-taking for conversation. *Language* 50 696–735. 10.2307/412243

[B52] Schiffrin D. (1987). *Discourse Markers.* Cambridge: Cambridge University Press. 10.1017/CBO9780511611841

[B53] Shokouhi H. Norwood C. Soltani S. (2015). *Evidential in Persian Editorials.* London: Sage Publications. 10.1177/1461445615578964

[B54] Shulman L. S. (1986). Those who understand: knowledge growth in teaching. *Educ. Res.* 15 4–14. 10.2307/1175860

[B55] Swales J. (1990). *Genre Analysis: English in Academic And Research Settings.* Cambridge: Cambridge University Press.

[B56] Swales J. (2001). Metatalk in american academic talk: the cases of point and thing. *J. Eng. Ling.* 29 34–54. 10.1177/00754240122005189

[B57] Trillo J. (2002). The pragmatic fossilization of discourse markers in non-native speakers of English. *Journal of Pragmatics* 34 769–784. 10.1016/S0378-2166(02)00022-X

[B58] Walsh S. (2011). *Exploring Classroom Discourse: Language in Action.* London: Taylor & Francis. 10.4324/9780203827826

[B59] Walsh S. (2006). *Analysing Classroom Discourse: A Variable Approach.* Oxon: Routledge. 10.4324/9780203015711

[B60] Walsh S. Morton T. O’Keeffe A. (2011). Analysing university spoken interaction: a corpus linguistics/conversation analysis approach. *Int. J. Corpus Ling.* 16 325–345. 10.1075/ijcl.16.3.03wal 33486653

[B61] Yang S. (2014). *Investigating Discourse Markers in Chinese College EFL Teacher Talk: A Multiple-Layered Analytical Approach Ph. D, Thesis.* Newcastle, UK: Newcastle University.

[B62] Zare J. Talakoli M. (2017). The use of personal metadiscourse over monologic and dialogic modes of academic speech. *Dis. Proc.* 54 163–175. 10.1080/0163853X.2015.1116342

[B63] Zhang L. (2017). *Classroom Discourse in Content-Based Instruction in Higher Education: A Focus on Teachers’ Use of Metadiscourse Ph. D, Thesis.* Hong Kong, China: University of Hong Kong.

[B64] Zhang L. Lo Y. Y. (2021). “EMI teachers’ use of interactive metadiscourse in lecture organization and knowledge construction,” in *Language use in English-Medium Instruction at University: International Perspectives on Teacher Practice*, eds Lasagabaster D. Doiz A. (Routledge), 56–79. 10.4324/9781003134534-4

